# Linker-specific monoclonal antibodies present a simple and reliable detection method for scFv-based CAR NK cells

**DOI:** 10.1016/j.omtm.2024.101328

**Published:** 2024-08-22

**Authors:** Katharina Schindler, Katharina Eva Ruppel, Claudia Müller, Ulrike Koehl, Stephan Fricke, Dominik Schmiedel

**Affiliations:** 1 Department for Cell and Gene Therapy Development, Fraunhofer Institute for Cell Therapy and Immunology (IZI), 04103 Leipzig, Germany; 2 Department of Preclinical Development and Validation, Fraunhofer Institute for Cell Therapy and Immunology (IZI), 04103 Leipzig, Germany; 3 Institute for Clinical Immunology, University of Leipzig, 04103 Leipzig, Germany; 4 Fraunhofer Cluster of Excellence Immune-Mediated Diseases, 60596 Frankfurt, Germany; 5 Medicine Campus MEDiC of the Technical University of Dresden at Klinikum Chemnitz gGmbH, 09116 Chemnitz, Germany

**Keywords:** cell therapy, gene therapy, CAR NK cells, CAR detection methods, flow cytometry, diagnostics, chimeric antigen receptor, single-chain variable fragment, scFv

## Abstract

Chimeric antigen receptor (CAR) T cell therapies have demonstrated significant successes in treating cancer. Currently, there are six approved CAR T cell products available on the market that target different malignancies of the B cell lineage. However, to overcome the limitations of CAR T cell therapies, other immune cells are being investigated for CAR-based cell therapies. CAR natural killer (NK) cells can be applied as allogeneic cell therapy, providing an economical, safe, and efficient alternative to autologous CAR T cells. To improve CAR research and future in-patient monitoring of cell therapeutics, a simple, reliable, and versatile CAR detection reagent is crucial. As most existing CARs contain a single-chain variable fragment (scFv) with either a Whitlow or a G4S linker site, linker-specific monoclonal antibodies (mAbs) can detect a broad range of CARs. This study demonstrates that these linker-specific mAbs can detect different CAR NK cells *in vitro*, spiked in whole blood, and within patient-derived tumor spheroids with high specificity and sensitivity, providing an effective and almost universal alternative for scFv-based CAR detection. Additionally, we confirm that linker-specific antibodies can be used for functional testing and enrichment of CAR NK cells, thereby providing a useful research tool to fast-track the development of novel CAR-based therapies.

## Graphical abstract

**Figure:**
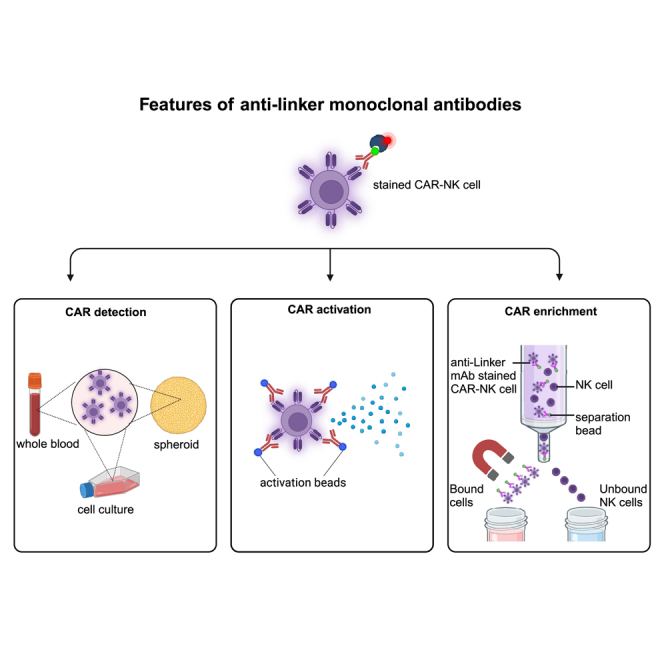

## Teaser

Schmiedel and colleagues evaluated linker-specific antibodies as a method for detecting CAR NK cells and compared them with existing methods. The data showed that linker-specific antibodies are a broadly applicable tool for detecting, activating, or purifying CAR NK cells, regardless of their specificity. This can accelerate their preclinical and clinical development.

## Introduction

Cell therapies become increasingly important in the treatment of various diseases, particularly cancer. Prominent examples are chimeric antigen receptor (CAR) T cell-based therapies, which show great clinical success in treating hematological disorders, such as B cell leukemias and lymphomas as well as multiple myeloma. ⁠^1^^,^^2^ Currently, there are six different CAR T cell products available on the market: Kymriah (tisagenlecleucel), Yescarta (axicabtagene ciloleucel), Breyanzi (lisocabtagene maraleucel), and Tecartus (brexucabtagene autoleucel) (targeting CD19 antigen), as well as Abecma (idecabtagene vicleucel) and Carvykti (ciltacabtagene autoleucel) (targeting B cell maturation antigen (BCMA)).⁠^3^^,^^4^ In preclinical research and clinical studies, CARnatural killer (NK) cells are gaining attention, as they show some potential advantages over CAR T cells.⁠^5^ NK cells exhibit a safe cytokine profile, resulting in fewer side effects, and can be transferred from healthy donors to patients (allogeneically), allowing use as an off-the-shelf product.⁠^6^ Cytokine-expanded, allogeneic NK cells have been used in numerous clinical studies,⁠^7^ , so far, only results from a single CAR NK cell study have been published to date.⁠^8^ However, these CD19-directed CAR NK cells yielded encouraging safety and efficacy data.⁠^8^ For the preclinical development of new therapies and the future monitoring of CAR NK cells in patients, a reliable and cost-effective detection method for CAR-engineered NK cells is crucial. However, such a method is currently unavailable. There are several types of antigen-binding domains that can be used for CAR design. The most commonly used are single-chain variable fragments (scFvs), which are derived from monoclonal antibodies. These consist of the variable heavy and light chains, which are connected by a linker. Another type of CAR utilizes the single-domain antibody fragments from camelid heavy-chain antibodies as the antigen-binding moiety. This offers advantages such as a smaller size and higher stability. Ligand-receptor pairs, such as interleukin-13 (IL-13) mutein-binding IL-13Rα2⁠,^9^ APRIL (a proliferation-inducing ligand),^10^^,^^11^ or NKG2D (natural killer group 2D),^12^ recognizing stress ligands can also be engineered into CARs, thus allowing the targeting of tumor-associated antigens without the need for antibodies. Given the current dominance of scFv-based CARs in both the clinic and the CAR NK cell field, our research focused on scFv-based CAR (hereafterreferred to as CAR) detection methods. Several detection methods for CARs exist, as protein L, which binds to the immunoglobulin kappa light chains, or polyclonal anti-immunoglobulin G (IgG) antibodies, which bind to the F(abʹ)_2_ fragment of an antibody and therefore detect CAR structures as well.^13^ However, these methods exhibit fluctuating specificity, and detection of CARs may be affected by the presence of Igs in human serum.^13^ Another detection method is the use of anti-idiotypic antibodies, which bind to the unique antigen-binding region (idiotope) of the CAR. This method is highly specific and sensitive, with low background staining compared to the aforementioned reagents,^14^ but it is noteworthy that the majority of anti-idiotype antibodies are difficult to obtain and are not commercially available. Flow cytometry detection with recombinant human (rh) BCMA or CD19 protein (recombinant human proteins [rh-proteins]) as CAR detection reagents currently dominates patient diagnostics in CAR T cell therapy.^15^^,^^16^ This method is highly precise and specific, but it is limited to only one specific target and therefore less suited for screening purposes in preclinical studies and for the detection of CARs against experimental antigens. As the vast majority of current CAR designs are based on an scFv as the binding domain, in which heavy and light chains are connected with distinct linker sequences,^17^ antibodies targeting these linkers may serve as almost universal scFv-based CAR detection reagents. Recently, two antibodies directed against either the glycine-serine linker sequence (G4S linker; GGGGSGGGGSGGGGS) or the Whitlow linker sequence 218^18^ (GSTSGSGKPGSGEGSTKG) were generated and commercialized.^19^

These linkers are widely used in research, in the majority of the clinically used CAR T cells (except for the Carvykti CAR, which has a unique nanobody structure) and in previously published clinical studies using CD19-directed CAR NK cells.⁠^6^^,^^8^^,^^20^ In this study, the sensitivity and specificity of linker-specific monoclonal antibodies (mAbs) for the detection of CAR NK cells were evaluated, as well as their potential to activate and purify CAR NK cells. To this end, the anti-G4S (a-G4S) mAb and anti-Whitlow (a-Whitlow) mAb were compared with other commonly used detection methods to improve preclinical and clinical development of CAR NK cells.

## Results

### CAR NK cells can be specifically detected with linker-specific mAbs *in vitro*

**Figure 1:**
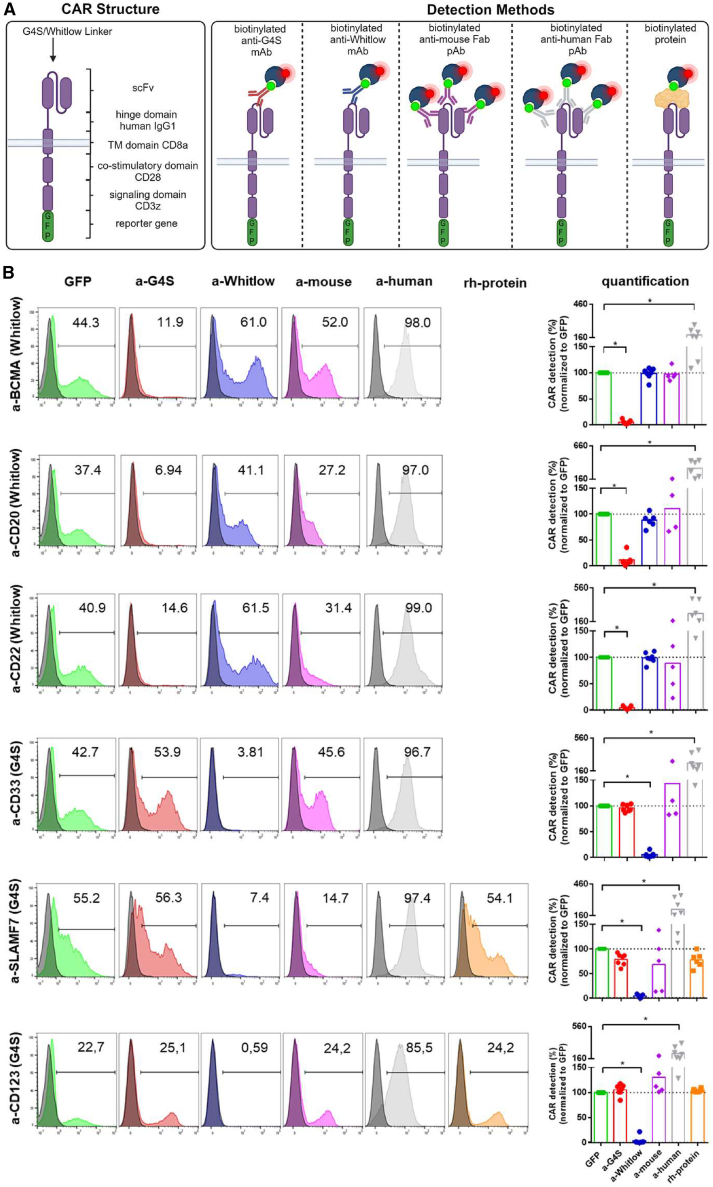
CAR NK cells can be specifically detected with CAR linker-specific mAbs *in vitro* (A) Schematic of the CAR structure (left box) and the different binding mechanisms of the CAR detection reagents used in the experiments (right box). The linker-specific mAbs bind to the respective linker sequence connecting the VL and VH chains. The polyclonal F(abʹ)_2_ antibodies bind to the F(abʹ)_2_ portion of the Ig. Antigen-specific rh-proteins bind to the paratope of the scFv. All antibodies used in the experiments are biotinylated and detected with fluorochrome-conjugated streptavidin in a second staining step. The figure was created with BioRender. (B) CAR expression on primary NK cells of healthy donors 3 days post transduction was analyzed via flow cytometry with different detection methods. Overlay histograms show GFP expression (green) or staining with a-G4S mAb (red), a-Whitlow mAb (blue), a-mouse F(abʹ)_2_ pAb (purple), a-human F(abʹ)_2_ pAb (gray), or rh-protein (orange) and a secondary antibody control (black), respectively. Data are shown as mean; *n* = 5–7. One representative donor is shown. Numbers indicate the percentage of gated cells. Quantification was done by normalization to GFP. Control, staining only with the secondary antibody. mAb, monoclonal antibody; pAb, polyclonal antibody. The *p* values were derived from one-way ANOVA followed by Dunnett’s multiple-comparisons test. ∗*p* ≤ 0.05.

To assess the suitability of linker-specific mAbs as CAR detection reagents, we transduced primary NK cells with different CAR constructs and detected CAR expression using various staining methods through flow cytometry (as illustrated in Figure 1). The study compared the a-G4S mAb and a-Whitlow mAb with three other commonly used detection strategies in research laboratories: anti-human F(abʹ)_2_ polyclonal antibody (a-human pAb) staining, anti-mouse F(abʹ)_2_ pAb (a-mouse pAb) staining, and recombinant human protein (rh-protein) staining. All CAR constructs contain GFP as a reporter gene (Figure 1A). Therefore, it is assumed that GFP expression is equivalent to CAR expression, and GFP is used as a reference. The positive and negative populations were separated by two peaks using the histogram setting. High transduction efficiencies of up to 50% were achieved for all CAR constructs (Figure 1B, one representative donor for each CAR construct). As shown in Figure 1B, both the a-Whitlow mAb as well as the a-G4S mAb could reliably detect all Whitlow-positive or G4S-positive CAR NK cells, respectively, compared to the GFP reference. Both linker-specific mAbs exhibit a clear positive signal, enabling a clear distinction between positive and negative populations. Both the a-G4S mAb and the a-Whitlow mAb showed no cross-reactivity with CARs containing the other linker sequence (Figure 1B). The staining with the a-mouse pAb displays high fluctuation within most groups, particularly for the humanized CAR scFvs (a-SLAMF7 and a-CD22), making reliable CAR detection impossible (Figure 1B). Additionally, the fluorophore signal appears dim, making it challenging to distinguish between positive and negative cell populations. The a-human pAb shows a significant increase in all CAR NK cells compared to the GFP standard (Figure 1B). The Fc receptor CD16 present on NK cells binds serum Igs, which react with the a-human pAb, leading to false positive results (Figure S1). The rh-protein staining is comparable to the GFP reference, which makes it a valid method for detecting CARs on primary NK cells (Figure 1B).

To further confirm these data and assess the suitability of linker-specific mAbs for CAR screening purposes on cell lines, we overexpressed diverse CARs in the HEK293T cell line. All CARs, containing either Whitlow or G4S linkers, were detected using the a-Whitlow mAb or a-G4S mAb, respectively, to the same extent as the GFP reference. This indicates a reliable method for detecting CARs, even on highly expressing cell lines (Figure S2). The a-mouse pAb, a-human pAb, and rh-protein stainings are also acceptable methods for detecting CARs on HEK293T cells. The only exception is the a-CD20 CAR, as all the different stainings exhibit high fluctuations. Notably, a-Whitlow mAb and a-human pAb stainings are significantly different from the GFP reference, indicating that none of the methods are suitable for detecting this specific CAR on HEK293T cells. However, overall, linker-specific mAbs can reliably detect CAR NK cells and CARHEK293T cells.

### The functionality of CARs expressed on NK cells can be assessed using linker-specific mAbs *in vitro*

**Figure 2:**
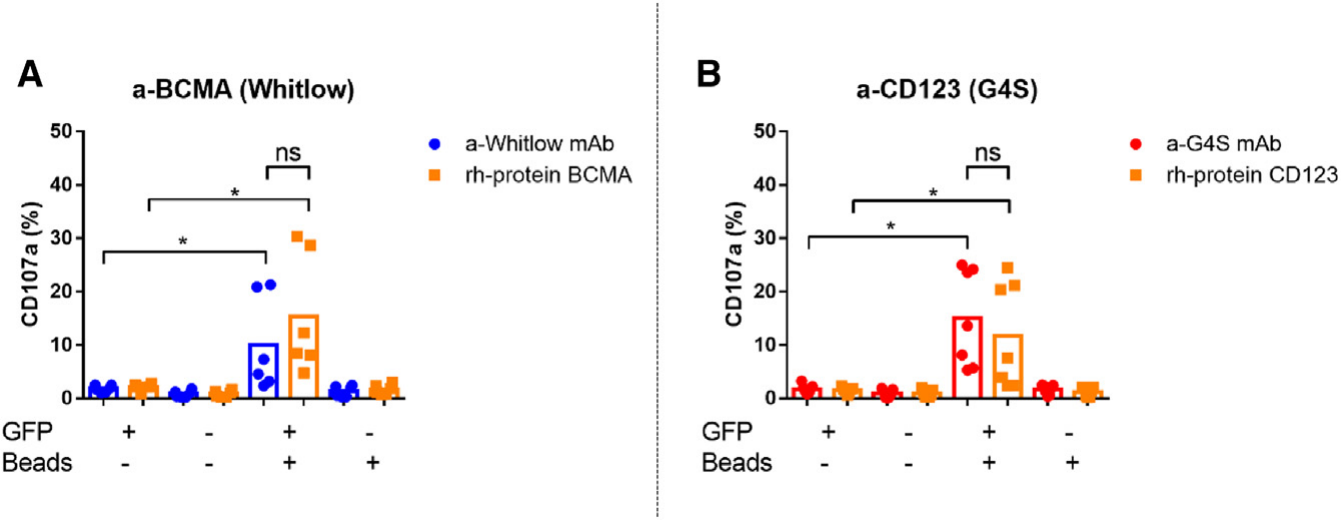
The functionality of CARs expressed on NK cells can be assessed using linker-specific mAbs *in vitro* CD107a expression of (A) a-BCMA and (B) a-CD123 CAR NK cells was analyzed with or without bead stimulation 3 days post transduction. Mean values of 6 independent healthy donor NK cells are shown. The *p* values were derived from two-tailed Student’s t test. ∗*p* ≤ 0.05; ns, not significant.

To evaluate whether linker-specific mAbs can activate NK cells in a CAR-dependent manner, a target-cell-independent activation assay was conducted. The CAR NK cells were labeled with the corresponding linker mAb or rh-protein as a control and co-incubated with and without anti-biotin MACSiBead particles. Subsequently, NK cell degranulation was determined by flow cytometry based on CD107a surface exposure. As anticipated, the unstimulated a-BCMA CAR NK cells stained with a-Whitlow mAb or rh-protein BCMA (GFP+, bead−) did not show surface expression of CD107a, indicating no activation of these cells (Figure 2A). In contrast, the CAR+ fraction that was incubated with beads (GFP+, bead+) showed a significant increase in CD107a expression compared to the unstimulated group, indicating strong activation (Figure 2A). There was no significant difference in CD107a expression between the linker mAb-stained and the rh-protein-stained CAR NK cells. This suggests that both staining methods are similarly suitable for activating CAR NK cells (Figure 2A). Similar results were observed for a-CD123 CAR NK cells stained with a-G4S mAb or rh-protein CD123 (Figure 2B). Accordingly, CAR-dependent NK cell activation can be triggered by both linker-specific antibodies.

### Linker-specific mAbs can be used for positive selection of CAR NK cells *in vitro*

**Figure 3:**
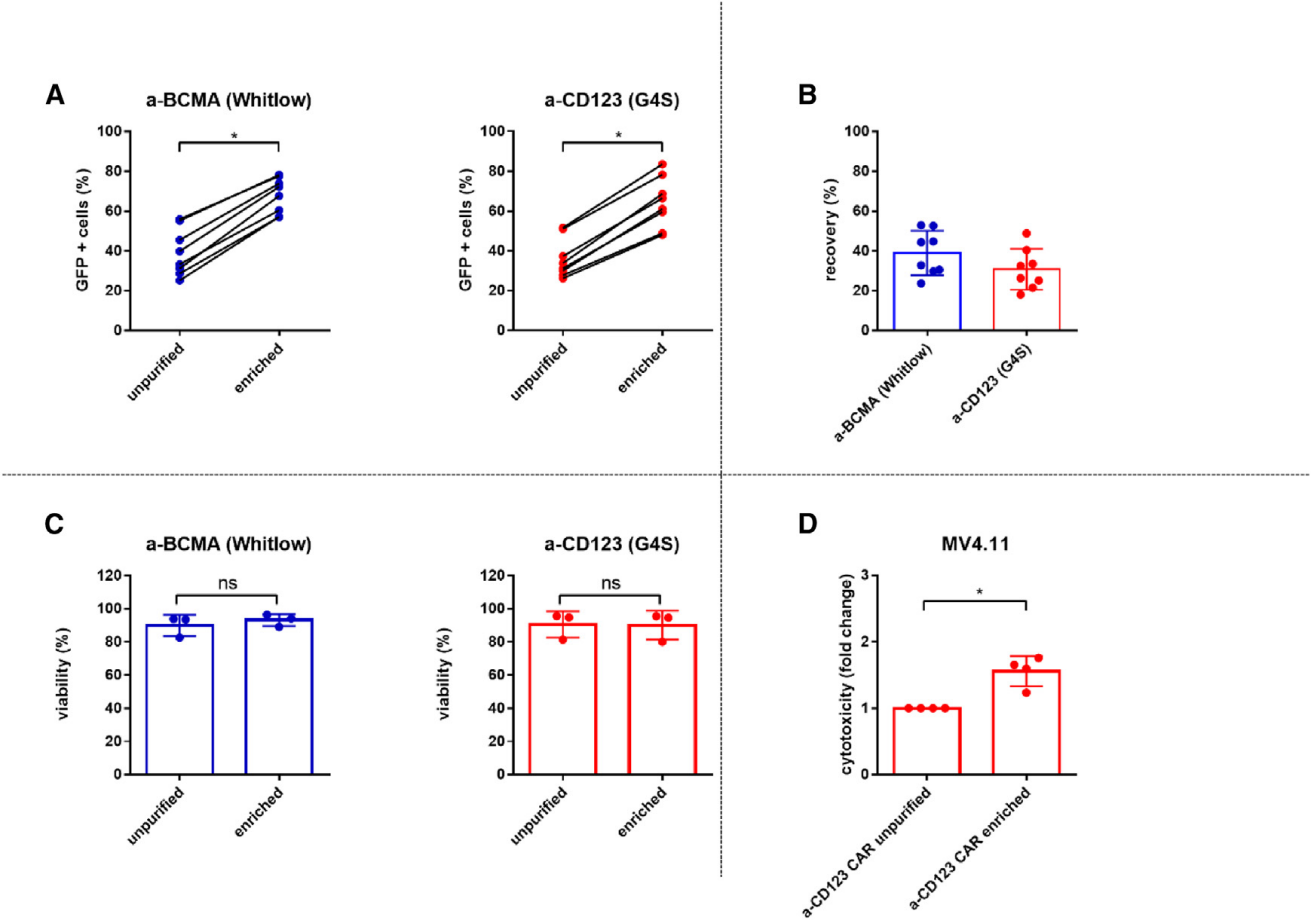
Linker-specific mAbs can be used for positive selection of CAR NK cells *in vitro* (A) CAR NK cells were stained with linker-specific mAbs 3–4 days post transduction, and GFP expression of CAR NK cells was analyzed by flow cytometry before (unpurified) and after (enriched) magnetic purification. (B) Scatterplots with bars show the recovery of CAR NK cells after the purification. (C) Viability was assessed with propidium iodide (PI) staining 2 days after purification by flow cytometry. (D) Cytotoxic activity was investigated by coculturing of unpurified or enriched a-CD123 CAR NK cells with CD123+ tumor cells (MV4.11) for 24 h at an E:T ratio of 0.125:1. Tumor cell death was determined by PI staining. To reduce donor variation, data were normalized to unpurified a-CD123 CAR NK cells and are shown as fold change. Data are represented as mean ± SD; *n* = 4–8. The *p* values were derived from two-tailed Student’s t test. ∗*p* ≤ 0.05.

To analyze the properties of linker-specific mAbs, we investigated their potential for enriching CAR NK cell suspensions. We used one Whitlow-containing CAR (a-BCMA) and one G4S-containing CAR (a-CD123) and performed flow cytometry analyses before and after the purification step. The labeling with the linker-specific antibodies and subsequent magnetic separation resulted in a significant, approximately 2-fold increase of CAR+ NK cells in the suspension (Figure 3A). The recovery rate of the CAR NK cells was only moderate, ranging from approximately 18% to 52% (Figure 3B). These results indicate that linker-specific mAbs can be used for positive selection of CAR NK cells, but a significant loss in CAR-NK cell numbers needs to be taken into consideration. However, the recovery may be further elevated by optimizing the choice of magnetic separation kits and the purification procedure. The viability of CAR NK cells was evaluated through propidium iodide (PI) staining of unpurified CAR NK cells and CAR NK cells that underwent the purification step. As illustrated in Figure 3C, the viability was found to be comparable between both groups, indicating that the purification of CAR NK cells did not affect viability. Furthermore, we investigated the functionality of non-enriched CAR NK cells and enriched CAR NK cells. As expected, enriched a-CD123 CAR NK cells killed significantly more CD123+ tumor cells (MV4.11) compared to unpurified a-CD123 CAR NK cells (Figure 3D). This enhanced killing activity is the effect of having more CAR NK cells in suspension due to the purification step and not due to enhanced activation, as shown in Figure S3. Only co-incubation with activation beads leads to enhanced CD107a surface expression, but not co-incubation with separation beads alone or CAR NK cells that have undergone the entire purification procedure (Figure S3). These results demonstrate that linker-specific antibodies can be used to enrich CAR NK cells and that these CAR NK cells are not activated or compromised in their viability; instead, the purified fraction mediates enhanced cytotoxic activity against tumor cells *in vitro*.

### Linker-specific mAbs can be used to detect CAR NK cells in whole-blood specimens as well as breast cancer tumor spheroids

**Figure 4:**
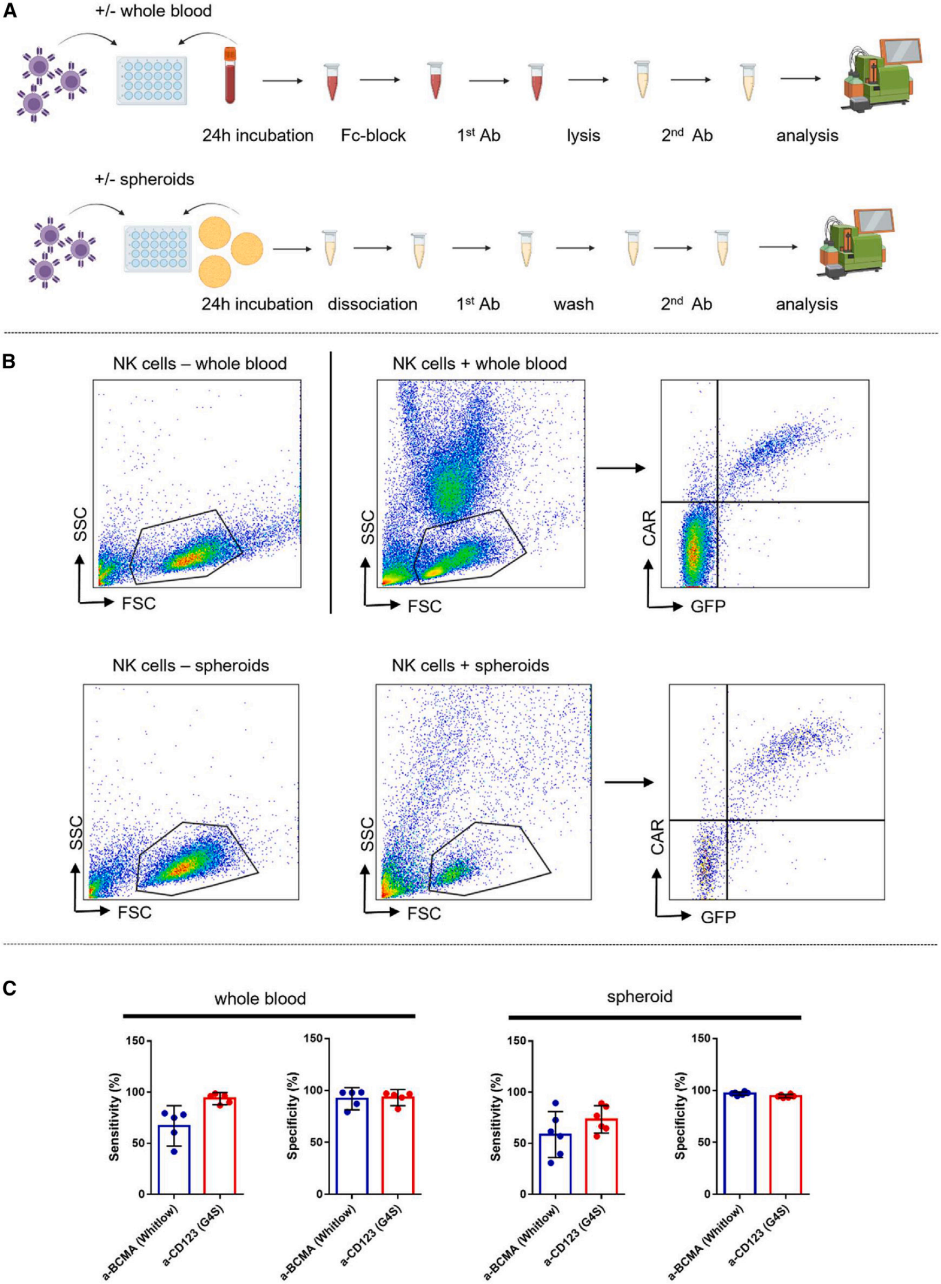
Linker-specific mAbs can be used to detect CAR NK cells in whole-blood specimens as well as breast cancer tumor spheroids (A) Schematic of the experimental procedure. 3 days post transduction, a-BCMA and a-CD123 CAR NK cells were co-cultured with or without whole blood or spheroids for 24 h and stained following a routine diagnostic protocol or the dissociation protocol. The figure was created with BioRender. (B) Gating strategy for the determination of sensitivity and specificity of linker-specific mAbs in whole-blood specimens and spheroid co-cultures. The NK cell population is identified according to the forward scatter/side scatter properties in a sample without blood/spheroids. This population is plotted in a graph with CAR-APC against GFP-fluorescein isothiocyanate. The top left quadrant contains the false positive cells (CAR+, GFP−), the top right quadrant contains the true positive cells (CAR+, GFP+), the bottom left quadrant contains the true negative cells (CAR−, GFP−), and the bottom right quadrant contains the false negative cells (GFP+, CAR−). One representative donor is shown. (C) Graphs represent sensitivity and specificity of linker-specific mAbs in whole blood or spheroids. Data are represented as mean ± SD; *n* = 5. For the spheroid assay, triplicates of 2 healthy NK cell donors are shown.

For future in-patient applications of CAR NK cells, it will be important that they are reliably detectable in whole blood or intratumorally to determine CAR NK cell counts and long-term persistence. To investigate whether linker-specific mAbs could be used for this purpose, CAR NK cells were co-cultured with or without whole blood or patient-derived tumor spheroids for 24 h. The next day, a routine diagnostic flow cytometry-based protocol was performed on the whole-blood suspensions, while the CAR NK cells co-cultured with the spheroids were first dissociated, followed by the staining protocol (Figure 4A). After setting the NK population gate, the cell populations were divided into false positive cells (CAR+, GFP−), true positive cells (CAR+, GFP+), false negative cells (CAR−, GFP+), and true negative cells (CAR−, GFP−) (Figure 4B) to determine sensitivity and specificity. Our results showed that a-BCMA CAR NK cells stained with the a-Whitlow mAb could be detected in whole blood with a sensitivity of 66.93% ± 15.87% and a specificity of 92.11% ± 8.55% (Figure 4C). The a-CD123 CAR NK cells, which were stained with the a-G4S mAb, showed a sensitivity of 93.61% ± 4.78% and a specificity of 93.02% ± 6.21% in whole-blood suspension (Figure 4C). When staining CAR NK cells co-cultured with spheroids, a-BCMA CAR NK cells were detected with a sensitivity of 58.51% ± 21.39% and a specificity of 97.04% ± 1.56%, whereas a-CD123 CAR NK cells were detected with a sensitivity of 73.34% ± 12.78% and a specificity of 94.57% ± 1.48% (Figure 4C). These findings suggest that linker-specific mAbs can be used to detect CAR NK cells in complex and multi-cellular suspensions *in vitro* and may be used for future in-patient diagnostics.

## Discussion

The use of CAR therapies in treating several hematological disorders has been highly successful in recent years, leading to increased interest in expanding their use to other malignancies as well as autoimmune diseases.^21^^,^^22^ As research and clinical studies of new CAR therapies continue to grow,^23^ there is a pressing need for a versatile and reproducible method of scFv-based CAR detection. This is important not only for clinicians to monitor CAR NK cell fate following administration but also for quality control of CAR products in manufacturing sites. For preclinical research, the assessment of the correct molecular design and expression of CAR constructs as well as effector functions are critical for the swift development and validation of novel CAR candidates.^13^ This study analyzed the potential of two linker-specific mAbs for their specificity and sensitivity in flow cytometry CAR detection, as well as their potential for activating and purifying CAR NK cells, in comparison to other commonly used detection reagents. Polyclonal a-mouse and a-human F(abʹ)_2_ antibodies are commonly used as CAR detection reagents in research due to their relatively low cost, high stability, and easy accessibility to academic labs.^13^ However, potential cross-reactivity with other IgG-like fragments can lead to unspecific binding and batch-to-batch variation of pAbs can greatly impact accurate CAR detection.^24^ This study shows that the a-mouse pAb can detect the CAR but with limited accuracy and reproducibility. This may be due to the polyclonal nature of the antibody, which can lead to varying degrees of binding to different areas of the target molecule. Furthermore, the a-mouse pAb demonstrates poor discrimination between CAR-expressing and negative cells in all plots, whereas the linker-specific mAbs provide a clear distinction between these two fractions, resulting in higher specificity. As scFvs are commonly humanized or developed to consist of fully human sequences to avoid immunogenicity,^25^ the a-mouse pAb cannot reliably detect these human(ized) scFvs. The a-SLAMF7 CAR and a-CD22 CAR, which contain a humanized scFvs, are poorly detected with the a-mouse pAb, highlighting the importance of an alternative CAR detection reagent. Whereas CART cells can be detected by a-human pAbs, this approach is not feasible for primary NK cells. The Fc receptor CD16 expressed on their cell surface binds Igs present in cell culture medium and in human serum. The a-human pAb binds to these antibodies, leading to false positive results. This was confirmed, as all a-human pAb-stained (CAR) NK cells exhibited a significant and extremely high artificial CAR signal, even in absence of a CAR. When also staining these NK cells with a CD16 marker, the untransduced control NK cells as well as the CAR NK cells show a double-positive signal. However, the a-human pAb was able to detect as many CAR-positive cells as the control (GFP) in cell types lacking Fc receptors, such as HEK293T cells, and have been shown previously to detect CARs on T cells.^24^ Nevertheless, due to the abovementioned limitations, neither anti-human nor anti-mouse IgG-specific antibodies are suitable for accurate and universal CAR detection on NK cells.

This study shows that both linker-specific mAbs can detect CARs on primary NK cells and the HEK293T cell line with high specificity and sensitivity, regardless of the antigen specificity of the respective CAR. This approach is cost effective, as only two reagents are needed to stain a variety of CARs. Additionally, the incorporation of this method into complex multi-parametric flow panels is easily manageable. The rh-proteins used for a-SLAMF7 (SLAMF7 protein) and a-CD123 (CD123 protein) were equally effective. However, they can only be used for a single specificity, and a specific reagent for each CAR has to be purchased and validated. Additionally, rh-proteins of only a few antigen targets are commercially available, making it challenging to use them for screening purposes or for the detection of CARs against experimental antigens. Additionally, some antigens cannot be readily expressed as soluble proteins; for example, multi-pass membrane proteins.^26^ Anti-idiotypic antibodies bind the variable regions of a particular scFv, rendering them highly specific. Jena et al. developed an anti-idiotype antibody with a detection limit of 0.1%.^14^ Due to their high specificity and low background staining, these antibodies have been used for CAR T cell detection in preclinical studies and clinical trials. However, the same disadvantages that rh-proteins have also arise for anti-idiotype antibodies. For future monitoring of CAR NK cells in patients, it is crucial to have a reliable CAR detection method that works well in multi-cellular and complex suspensions, like in blood or in tissues. The analyses demonstrated a high sensitivity in detecting CAR NK cells in whole blood of approximately 67%–94% for both linker-specific mAbs, indicating that 67%–94% of the true CAR+ cells could be specifically identified. Furthermore, the data exhibit a high specificity (approximately 90%–99%) in whole-blood specimens, indicating minimal non-specific binding. These data are comparable to the specificity of the BCMA detection reagent from Miltenyi Biotec (Germany) used in the experiments by Reichman et al.^27^ They detected a sensitivity of about 83% and specificity of about 99.96%. To analyze whether CAR NK cells could be also detected within tumor tissue, they were co-cultured with patient-derived triple-negative breast cancer spheroids. 3D *in vitro* models, like cancer spheroids, better mimic the tumor microenvironment of solid tumors than 2D monolayer cultures.^28^ When staining with linker-antibodies, CAR NK cells could be detected with a sensitivity ranging from approximately 59%–73% and a specificity ranging from 95%–97%. The slightly reduced sensitivity compared to whole-blood staining can be due to the dissociation procedure. However, this procedure could be further improved by optimizing this protocol. Taken together, these data emphasize the potential of the linker-specific mAbs being used as a simple and reliable detection reagent in clinics as well as in preclinical research.

Additionally, we show that the linker-specific antibodies can be valuable research tools to enrich CAR NK cells and test their functionality in bead-based, target-cell-independent assays. To assess CAR functionality in NK cells, assays that activate CAR signaling specifically are superior to commonly used target cell-based assays, as target cells trigger a variety of activating and inhibitory NK cell receptors that determine natural cytotoxicity. These interactions trigger additional signaling pathways, possibly disturbing the readout of CAR-mediated effects.^29^ By titrating antibodies suitable for CAR activation, diverse antigen densities can be simulated, and the responsiveness of different CAR constructs may be elegantly compared. The functionality of the enriched CAR NK cells was consistent with expectations, as the higher CAR expression led to enriched killing of the target cells, while it did not affect the viability or activation status compared to non-enriched CAR NK cells. The purification assay and the target-cell-independent activation assay both utilize beads, but these beads differ in their characteristics, leading to the observed results. The anti-biotin MACSiBead particles from Miltenyi (referred to as activation beads) employed in the target-cell-independent activation assay are specifically designed for the activation of cells. For the purification assay, streptavidin nanobeads (referred to as separation beads) were used. These nanobeads are smaller in size and allow for less cell perturbation, such as cell activation.^30^ These attributes were the rationale behind their selection for the magnetic separation process, and we could show that they indeed did not affect the characteristics of CAR NK cells.

One important limitation of our study is that all CAR constructs used contain an IgG1-derived hinge domain and are designed using a variable heavy (VH) chain followed by a variable light (VL) chain configuration (VH-VL). These features may impact scFv confirmation, and differences in linker accessibility may, in turn, affect the binding abilities of the linker-specific antibodies. To uncover the potential limitations of linker-specific mAbs as CAR detection reagents, future studies should consider implementing different CAR designs or configurations. In conclusion, this work introduces CAR linker-specific mAbs as valuable research tools allowing faster development of novel CAR NK therapeutics. Additionally, they represent a promising detection method for a cost-efficient and versatile clinical in-patient monitoring of CAR NK cell-based therapies.

## Material and methods

### Construction of gammaretroviral vectors

All retroviral CAR constructs are second-generation constructs containing the respective scFv against the target BCMA (a-BCMA, C12A3.2, sequence 4 of a patent^31^), SLAMF7 (a-SLAMF7, huLuc63^32^), CD20 (a-CD20, rituximab^33^), CD22 (a-CD22, inotuzumab^34^), CD33 (a-CD33^35^), and CD123 (a-CD123, clone number 26292^36^). All CARs were constructed in the following pattern from the 5′ end to the 3′ end: CD8a signal peptide, scFv with VH-VL configuration, a human IgG1 hinge, a CD8a transmembrane domain, and signaling domains derived from CD28 and CD3zeta receptors.^37^ The CARs were designed with identical starts and ends of framework regions flanking the complementarity-determining regions in order to be amplified with the same set of primers for facilitated cloning. The sequences of all scFvs are listed in Table S1. Furthermore, all constructs contain GFP as a reporter gene and either G4S (SLAMF7, CD33, and CD123) or Whitlow (BCMA, CD20, and CD22) as a linker. The a-BCMA CAR, a-CD33 CAR, and a-CD123 CAR have a P2A site between the CAR and GFP, while all other CARs are directly fused to GFP. These constructs were cloned into the gammaretroviral pBullet vector (kindly provided by Reno Debets, Erasmus University Medical Center).^38^

### Generation of human CAR NK cells

NK cells were isolated from peripheral blood obtained from healthy donors at the Institute for Transfusion Medicine of the University Clinic in Leipzig, Germany (ethics vote number 327/22-ek) using the RosetteSep Human NK Cell Enrichment Cocktail (STEMCELL Technologies, Canada). The whole-blood sample was incubated with the RosetteSep Cocktail at room temperature for 10 min and then centrifuged with density gradient medium. Following blood lysis and several washing steps, the cells were cultured in NK MACS culture medium (Miltenyi), 5% human serum (Sigma-Aldrich, USA), 500 U/mL IL-2, and 140 U/mL IL-15 (PeproTech, USA). The cells were then incubated at 37°C and 5% CO_2_ for 7 days. To transduce the NK cells with different CAR constructs, 250,000 NK cells per well were seeded in a 24-well plate. 10 μg/mL Vectofusin-1 (Miltenyi) was used as a transduction enhancer, and 500 μL of viral supernatant was added per well. To achieve better transduction performance, the plate was centrifuged at 37°C for 1 h (400 or 1,200 × *g*), followed by the addition of 500 μL of fresh complete culture medium. The plate was then statically incubated at 37°C and 5% CO_2_. All experiments were conducted 3–6 days after transduction using flow cytometry (MACSQuant10, Miltenyi).

### Retrovirus production and transient transfection of HEK293T cells

Gammaretroviral particles were generated using HEK293T cells. 75,000 cells were seeded in one well of a 12-well plate, 48 h prior to transfection. On the day of transfection, the retroviral transfer plasmids encoding CAR sequences were co-transfected with the viral packaging plasmids pHIT60 and the baboon envelope (BaEV) at a 1:1.5 ratio (0.154 μg pHIT60 and 0.230 μg BaEV) using the TransIT transfection reagent (Mirus Bio, USA). After 48 h, the supernatant containing the viral particles was harvested and immediately used to transduce primary NK cells.

For the CAR staining on HEK293T cells, transient transfection of the CAR-encoding transfer plasmid was performed. After 48 h, the supernatant was discarded, and the HEK293T cells were used for cell-surface staining.

### Flow cytometry-based detection of different CAR constructs

Three days after transduction, all cells were stained for CAR expression using the following primary biotinylated antibodies: a-human F(abʹ)_2_ antibody (Biotin-SP [long spacer] AffiniPure F(abʹ)₂ fragment goat anti-human IgG, Jackson ImmunoResearch Europe), a-mouse F(abʹ)_2_ antibody (Biotin-SP [long spacer] AffiniPure F(abʹ)₂ fragment goat anti-mouse IgG, Jackson ImmunoResearch Europe), a-Whitlow mAb (Whitlow/218 linker [E3U7Q] rabbit mAb [biotinylated] 32523, Cell Signaling Technology), or a-G4S mAb (G4S linker [E7O2V] rabbit mAb [biotinylated] 17621, Cell Signaling Technology). The CD123 CAR construct and SLAMF7 CAR construct were additionally stained with either a-CD123 protein (biotinylated human IL-3Rα/CD123 protein, His, Avitag, Thermo Fisher Scientific, Germany) or a-SLAMF7 protein (biotinylated human SLAMF7, Avitag, His tag [SL7-H82E0], Avantor, Germany), respectively. After incubating the cells for 30 min at 4°C, they were washed once and then incubated for an additional 10 min at 4°C with the secondary antibody (streptavidin, APC, Miltenyi), followed by two washing steps. The MACSQuant10 analyzer (Miltenyi) was used to analyze the cells.

### Positive selection assay of CAR NK cells

For the purification assay, a-BCMA CAR NK cells and a-CD123 CAR NK cell suspensions were stained with either the a-Whitlow mAb or a-G4S mAb, respectively, as described previously. The CAR NK cell suspensions were then mixed with streptavidin nanobeads (MojoSort Human NK Cell Isolation Kit, BioLegend, USA) and incubated on ice for 15 min. The cells were subsequently washed, resuspended in 2.5 mL of 1× MojoSort buffer, and placed in the MojoSort magnet (BioLegend) for 5 min. The unlabeled cells were removed, and the CAR NK cells, which were labeled with nanobeads, were resuspended in MojoSort buffer and subsequently analyzed. Recovery was calculated using the following formula:

$$Recovery=\left(\frac{\#\;of\;cells\;in\;the\;purified\;sample}{\#\;of\;cells\;available\;for\;purification}\right)\cdot 100$$

### Flow cytometry-based target-cell-independent activation assay

The a-BCMA CAR NK cells, which contain the Whitlow linker, were stained with the a-Whitlow mAb or BCMA detection reagent (kindly provided by Maik Friedrich, Institute of Clinical Immunology, University of Leipzig, Germany). Similarly, the a-CD123 CAR NK cells, which contain the G4S linker, were stained with the a-G4S mAb or the CD123 recombinant protein, as described previously. Following staining, the CAR NK cells were co-incubated with or without anti-biotin MACSiBead particles (Miltenyi) in a 96-well plate for 2 h at 37°C and 5% CO_2_. After the incubation period, anti-CD56-APC-Vio770 and anti-CD107a-PE-Vio770 (LAMP-1) antibodies were added. The mixture was incubated for an additional 2 h before direct analysis with the MACSQuant10 analyzer (Miltenyi).

### Detection of CAR NK cells in whole blood

Fresh EDTA blood was obtained from healthy donors at the Institute for Transfusion Medicine of the University Clinic of Leipzig, Germany (ethics vote number 327/22-ek). 500,000 a-BCMA CAR NK cells and a-CD123 CAR NK cells were seeded in a 24-well plate and incubated with or without (control group) 500 μL of EDTA-treated blood for 16 h at 37°C and 5% CO_2_. After the incubation period, the suspension of cells and blood was collected and incubated with FcR blocking reagent (Miltenyi) for 10 min at 4°C. The suspension was then stained with primary antibodies (Whitlow or G4S linker), respectively. Following staining, erythrocyte lysis was performed by adding ACK (Ammonium-Chloride-Potassium) lysis buffer (Thermo Fisher Scientific, USA) for 10 min at room temperature. Finally, the suspension was treated with the secondary antibody, streptavidin-APC, for 10 min at 4°C. The cells were then washed twice and analyzed using flow cytometry (MACSQuant10, Miltenyi). To determine sensitivity and specificity, the following equations were used:

$$Sensitivity=\frac{true\;positive\;\left(CAR+,GFP+\right)}{true\;positive\;\left(CAR+,GFP+\right)+false\;negative\;\left(CAR-,GFP+\right)}$$

$$Specificity=\frac{true\;negative\;\left(CAR-,GFP-\right)}{true\;negative\;\left(CAR-,GFP-\right)+false\;positive\;\left(CAR+,GFP-\right)}$$

### Generation of spheroids and detection of CAR NK cells in spheroids

The triple-negative breast cancer sample was obtained from one chemotherapy-treated patient, with their informed consent, at the Clinic Braunschweig upon acceptance of the Ärtzekammer Niedersachsen (authorization Grae/231/2018). Patient biopsies were placed in a sterile 2-mL tube containing cell culture medium (DMEM:F12 [PanBiotech, Germany] with 20% fetal bovine serum [Merck, Germany], 0.023 U/mL insulin, 0.5 μg/mL hydrocortisone, and 10 ng/mL human epidermal growth factor (hEGF) [all from Sigma-Aldrich, Germany]) at 4 °C for transport. Upon arrival, biopsy samples were minced in 1- to 3-mm^3^ sections and cultured in a 6-well plate. The spheroids were generated from expanded primary tumor cells at passage 3. For the spheroids, 50 μL Matrigel matrix (9.9 mg/mL; Corning, Germany) was placed in a 96-well plate (flat bottom) and incubated at 37°C for 30 min. The tumor cells were washed once with PBS and trypsinized. The cells were resuspended with culture medium to adjust the final cell density to 1 × 10^5^ cells/mL. 50 μL of the prepared cell suspension was plated into each well of the pre-coated 96-well plate and incubated at 37°C for 30 min. In the final step, 100 μL of 10% Matrigel matrix (final concentration, 0.8 to 1.1 mg/mL; diluted with pre-chilled culture medium) was added to the culture. On the day of the experiment, 50,000 a-BCMA CAR NK cells and a-CD123 CAR NK cells were added to the spheroids and incubated for 24 h at 37°C and 5% CO_2_. After the incubation period, only the spheroids were carefully transferred into a new 96-well U-bottom plate, washed once, and treated with Accutase (Thermo Fisher Scientific, USA) for 10 min at 37°C to dissolve the spheroids. After a second washing step, the suspension was stained with primary antibodies (a-Whitlow mAbs or a-G4S mAbs, respectively), followed by treatment with the secondary antibody, streptavidin-APC, for 10 min at 4°C. The cells were then washed twice and analyzed using flow cytometry (MACSQuant10, Miltenyi). To determine sensitivity and specificity, the same equations were used as described under Detection of CAR NK cells in whole blood.

### Assessment of viability and functionality of CAR NK cells

For the assessment of viability and functionality, the enriched CAR NK cells were seeded in a 96-well plate and incubated overnight at 37°C and 5% CO_2_. The next day, the MV4.11 cell line was stained with CellTrace Violet (Thermo Fisher Scientific, USA) in order to be able to discriminate between a-CD123 CAR NK cells and tumor cells. Briefly, MV4.11 cells were stained with the reagent at a concentration of 0.5 μM for 20 min at 37°C, followed by adding five times the volume of serum-containing cell culture medium (RPMI 1640 medium, without phenol red (Thermo Fisher Scientific, USA) with 10% fetal calf serum (Bio&Sell, Germany) to stop the reaction. After the labeling, MV4.11 tumor cells were co-cultured with either unpurified or enriched a-CD123 CAR NK cells at an effector-to-target (E:T) ratio of 0.125:1 and incubated for 24 h at 37°C and 5% CO_2_. After the incubation, the assay was analyzed using flow cytometry (MACSQuant10, Miltenyi) with PI solution (Miltenyi) applied directly before each measurement to analyze cell death. To assess the viability of CAR NK cells, PI on the CellTrace-negative, GFP-positive population was quantified. To assess viability of tumor cells, PI was analyzed on the CellTrace-positive fraction.

### Statistical analysis

Data are presented as mean or as mean ± standard deviation (SD). All statistical analyses were performed using GraphPad Prism v.7.0 for Windows (GraphPad, San Diego, CA, USA). Differences between two groups were evaluated by two-tailed Student’s t test. One-way ANOVA was used to compare three or more groups, followed by Dunnett’s multiple-comparisons test unless otherwise stated. *p* < 0.05 was considered statistically significant.

## Data and code availability

All relevant data are included in the paper. Raw data are available upon request from the corresponding author.

## Acknowledgments

We would like to thank Dr. Maria-Irene Hainich from the Clinic Braunschweig and the Fraunhofer CIMD (Cluster of Excellence Immune-Mediated Diseases) biobank for the collection, preparation, and provision of samples. This work was supported by the Fraunhofer Internal Programs under Grant Attract 131-600004, by the 10.13039/501100002347German Federal Ministry of Education and Research under funding code 03ZU1111CA as part of the Clusters4Future cluster SaxoCell, by the Fraunhofer CIMD, and the “CAR Factory” consortium funded by the 10.13039/501100005972German Cancer Aid.

The responsibility for the content of this publication lies with the author.

## Author contributions

Conceptualization, D.S.; data curation, K.S.; formal analysis, K.S.; funding acquisition, D.S.; investigation, K.S.; methodology, K.S., C.M., and K.R.; project administration, D.S.; resources, U.K. and S.F.; supervision, D.S.; validation, D.S. and K.S.; visualization, K.S.; writing – original draft, K.S. and D.S.; writing – review and editing, U.K. and S.F.

## Declaration of interests

The authors declare no competing interests.

## Supplemental information

Document S1. Figures S1–S3 and Table S1

Document S2. Article plus supplemental information
