# Supplementary material for: Linker-specific monoclonal antibodies present a simple and reliable detection method for scFv-based CAR NK cells
Source: Mol Ther Methods Clin Dev. 2024 Aug 22;32(3):101328. doi: 10.1016/j.omtm.2024.101328 (PMC11403257; doi:10.1016/j.omtm.2024.101328)
Supplement: Document S1. Figures S1–S3 and Table S1 [file mmc1.pdf]

OMTM, Volume *32*

Supplemental information

Linker-specific monoclonal antibodies present a simple and reliable detection method for scFv-based CAR NK cells

Katharina Schindler, Katharina Eva Ruppel, Claudia Müller, Ulrike Koehl, Stephan Fricke, and Dominik Schmiedel

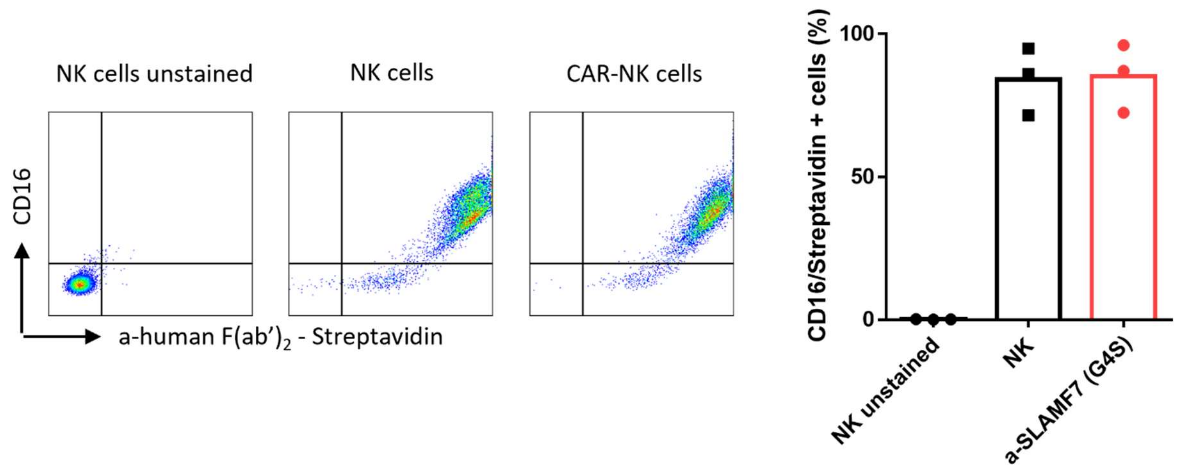

<u>Figure S1: Anti-human F(ab')$_2$ antibody shows cross reactivity with Fc receptors on NK cells.</u> Untransduced cells (NK) and CAR-NK cells (here a-SLAMF7 CAR-NK cells) were stained for CD16 (Fc receptor) and anti-human F(ab')$_2$ pAb. Dot plots show the staining for one representative donor. Bar plots depict the percentage of CD16/Streptavidin positive cells. Mean values are shown from n = 3 healthy donors.

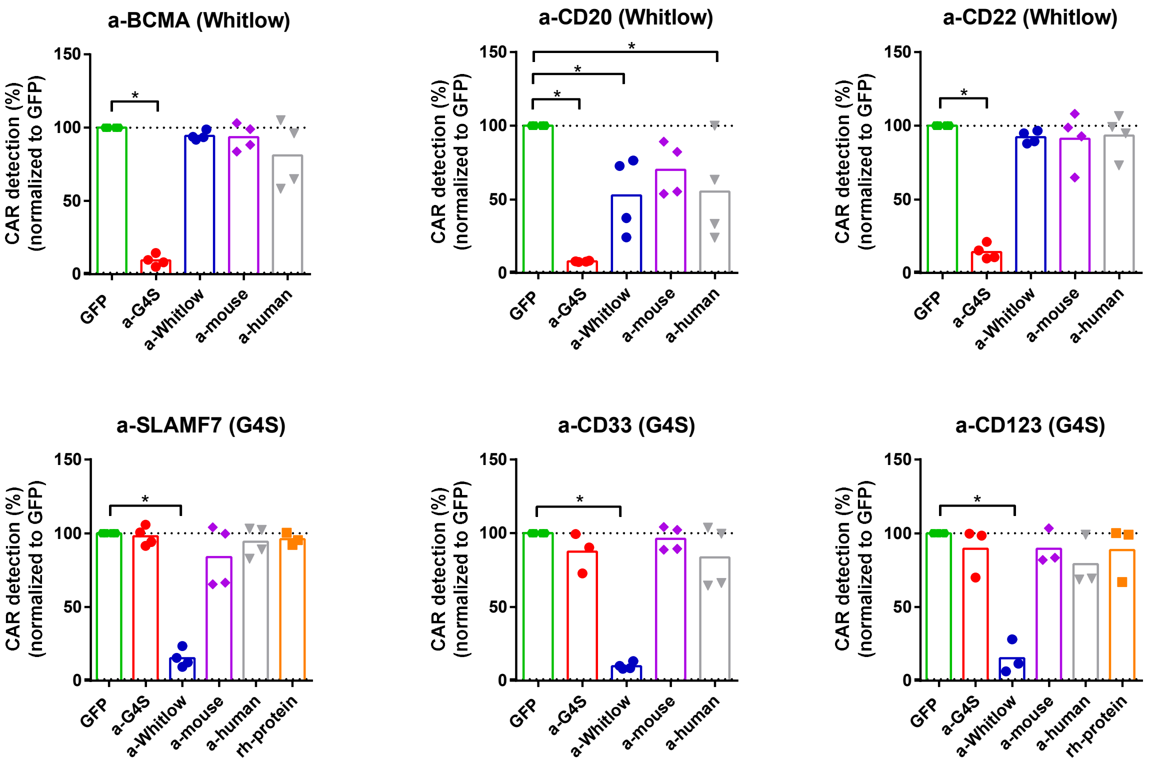

Figure S2: Linker-specific mAbs can be used for CAR detection in HEK293T cells. Different CAR detection reagents were compared on HEK293T cells and were analyzed via flow cytometry. Graphs represent the percentage of CAR-HEK293T cells produced from 3-4 independent experiments. Data are shown as mean and P-values derived from one-way ANOVA followed by Dunnett's multiple comparison test. * $P \leq 0.05$.

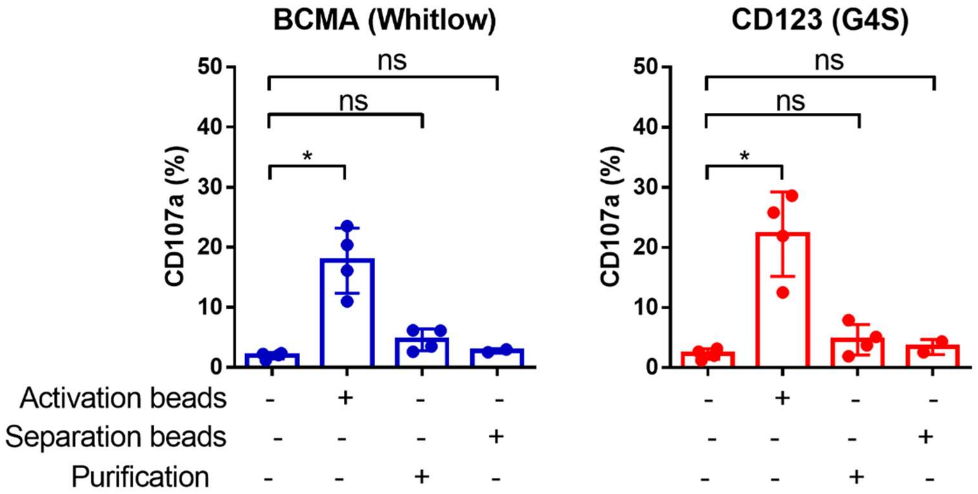

<u>Figure S3: Separation beads used in the purification protocol do not lead to activation of CAR-NK cells.</u> CD107a expression of unpurified CAR-NK cells co-incubated either with activation beads or separation beads, or purified CAR-NK cells is shown. Data are represented as mean ± S.D.; n = 4. P-values derived from one-way ANOVA followed by Sidak's multiple comparison test, with a single pooled variance. * $P \leq 0.05$; ns = not significant.

Table S1: Amino acid sequences of scFv's of the CAR constructs. Linker sequences are highlighted.

CAR construct	Linker	Amino acid sequences
a-BCMA CAR	Whitlow	MALPTALLLPLALLLHAARPQVQLVQSGPELKKPGETVKISCKASGYTFTDYSINWVKRAPGKGLKWMGWINTETREPAYAYDFRGRFAFSLETSASTAYLQINNLKYEDTATYFCALDYSYAMDYWGQGTSVTVSS**GSTSGSGKPGSGEGSTKG**DIVLTQSPPSLAMSLGKRATISCRASESVTILGSHLIHWYQQKPGQPPTLLIQLASAVQTGVPARFSGSGSRTDFTLTIDPVEEDDVAVYYCLQSRTIPRTFGG
a-CD20 CAR	Whitlow	MALPVTALLLPLALLLHAARPQVQLVQPGAELVKPGASVKMSCKASGYTFTSYNMHWVKQTPGRGLEWIGAIYPRNGDTSYNQKLKGKATLTADKSSSTAYMQLSSLTSEDSAVYYCARSTYYGGDWYFNVWGAGTTVTVSA**GSTSGSGKPGSGEGSTKG**QIVLSQSPAILSASPGEKVTMTCRASSSVSYIHWFQQKPGSSPKPWIYATSNLASGVPVRFSGSGSGTSYSLTISRVEAEDAATYYCQQWTSNPPTFGGGTKVEIK
a-CD22 CAR	Whitlow	MALPVTALLLPLALLLHAARPQVQLVQSGAEVKKPGASVKVSCKASGYRFTNYWIHWVRQAPGQGLEWIGGINPGNNYATYRRKFQGRVTMTADTSTSTVYMELSSLRSEDTAVYYCTREGYGNYGAWFAYWGQGTLVTVSS**GSTSGSGKPGSGEGSTKG**DVQVTQSPSSLSASVGDRVTITCRSSQSLANSYGNTFLSWYLHKPGKAPQLLIYGISNRFSGVPDRFSGSGSGTDFTLTISSLQPEDFATYYCLQGTHQPYTFGQGTKVEIK
a-SLAMF7 CAR	G4S	MALPVTALLLPLALLLHAARPEVQLVESGGGLVQPGGSLRLSCAASGFDFSRYWMSWVRQAPGKGLEWIGEINPDSSTINYAPSLKDKFIISRDNAKNSLYLQMNSLRAEDTAVYYCARPDGNYWYFDVWGQGTLVTVSS**GGGGSGGGGSGGGGS**DIQMTQSPSSLSASVGDRVTITCKASQDVGIAVAWYQQKPGKVPKLLIYWASTRHTGVPDRFSGSGSGTDFTLTISSLQPEDVATYYCQQYSSYPYTFGQGTKVEIK
a-CD33 CAR	G4S	MALPVTALLLPLALLLHAARPQVQLQQPGAEWKPGASVKMSCKASGYTFTSYYIHWIKQTPGQGLEWVGVIYPGNDDISYNQKFQGKATLTADKSSTTAYMQLSSLTSEDSAVYYCAREVRLRYFDVWGQGTTVTVSS**GGGGSGGGGSGGGGS**EIVLTQSPGSLAVSPGERVTMSCKSSQSVFFSSSQKNYLAWYQQIPGQSPRLLIYWASTRESGVPDRFTGSGSGTDFTLTISSVQPEDLAIYYCHQYLSSRTFGQGTKLEIK
a-CD123 CAR	G4S	MALPQVQLQQPGAELVRPGASVKLSCKASGYTFTSYWMNWVKQRPDQGLEWIGRIDPYDSETHYNQKFKDKAILTVDKSSSTAYMQLSSLTSEDSAVYYCARGNWDDYWGQGTTLTVSS**GGGGSGGGGSGGGGS**DVQITQSPSYLAASPGETITINCRASKSISKDLAWYQEKPGKTNKLLIYSGSTLQSGIPSRFSGSGSGTDFTLTISSLEPEDFAMYYCQQHNKYPYTFGGGTKLEIK
